# Diabetes Second-Line Medication Prescription Patterns in Costa Rica and Panama: Evidence From the DISCOVER Registry

**DOI:** 10.7759/cureus.16060

**Published:** 2021-06-30

**Authors:** Chih H Chen-Ku, Pilar Grimaldo de Sucre, Mary Vinocour, Luis C Ramírez-Zamora, Fernando Andrés-Jiménez, Claudio Slon-Hitti, Alejandro Cob, Guiselle Rodríguez

**Affiliations:** 1 Endocrinology, Clinica Los Yoses, San Pedro, CRI; 2 Internal Medicine, Hospital Aquilino Tejeira, Penonomé, PAN; 3 Endocrinology, Clinica Via San Juan, Tres Ríos, CRI; 4 Endocrinology, Clínica Los Yoses, San Pedro, CRI; 5 Endocrinology, Clínica Serenidad, San José, CRI; 6 Family Medicine, Private Practice, Heredia, CRI; 7 Internal Medicine, Private Practice, Penonomé, PAN

**Keywords:** complications, control, central america, pharmacologic treatment, diabetes mellitus

## Abstract

Aim: This study aimed to describe the prescription patterns of second-line medications for patients with diabetes from selected centers in Costa Rica and Panama.

Methods: DISCOVER is a registry of patients with type 2 diabetes switching from first- to second-line medications. We analyzed medication choice and the reasons to switch for each country.

Results: A total of 219 patients were included during 2014-2016, 127 in Costa Rica and 92 in Panama. The most frequently prescribed first-line medication was metformin, followed by sulphonylureas in Panama, and a combination of metformin and dipeptidyl peptidase-4 inhibitor (iDPP4) in Costa Rica. DPP4 inhibitors plus metformin was the most commonly prescribed second-line medication, followed by metformin combined with sodium-glucose transport protein-2 inhibitor (iSGLT2) in Costa Rica and iDPP4 in monotherapy in Panama. The main reason to switch being efficacy. When choosing the second-line medication, the main reasons behind the switch were efficacy, weight loss, and hypoglycemia risk in both countries (tolerability being also common in Panama).

Conclusions: According to the DISCOVER registry, in Costa Rica and Panama, efficacy is the most common reason to switch to second-line medication. Metformin plus iDPP4 was the most commonly prescribed agent.

## Introduction

The prevalence of type 2 diabetes has increased worldwide from 8.3% in 2013 to 9.3% in 2019 [1,2]. This increase has been higher in lower- to middle-income countries compared to high-income ones. By 2025, in Central America (including Costa Rica and Panama), there will be more than two million patients with diabetes, a 162% increase from 1995 [3,4]. The increase in these two countries is expected to be 8.1% and 10.5%, respectively [5]. A recent meta-analysis showed that diabetes increased all cause and cardiovascular mortality to a greater extent in Latin American countries compared to high-income ones [6].

Diabetes treatment includes behavioral and pharmacological approaches. There are several medications currently approved, and the choice depends on availability and healthcare system type. Costa Rica and Panama have both public and private healthcare systems. In Costa Rica, the one public system is the Caja Costarricense del Seguro Social (Social Security System) that covers 95% of the population. In Panama, two public systems (not mutually exclusive) are available, the Ministerio de Salud (Health Ministry, 74.2% coverage) and Caja de Seguro Social (Social Security, 75% coverage). Private care is available in both countries and paid through insurance or by the patient. Regarding diabetes treatment, different medications are available depending on each country and system type.

Globally, it is estimated that only 23% (30% in Latin America) of all patients with diabetes achieve set targets [7]. However, for physicians treating them, it is now a challenge to choose the treatment scheme suitable for each patient. Availability, affordability, patients’ preference, side effects, and success rates, are amongst a myriad of determinants when choosing the appropriate medication [8]. Current guidelines suggest tailoring treatment choices according to patient characteristics [9]. However, in the real world, little is known about the reasons that drive physicians to stop and select an antidiabetic drug.

DISCOVER is a worldwide study designed to provide real-world data on how first- and second-line type 2 diabetes medications are prescribed. According to DISCOVER Latin America, there is great heterogeneity between countries regarding when to start and how to select the second-line medications [10]. In that paper, Costa Rica and Panama were reported together as Central America and compared to other countries in Latin America [10]. Given that Costa Rica and Panama are two countries with different healthcare systems and patient characteristics, we sought to describe prescription patterns using data from these local DISCOVER databases.

This article was previously published as a preprint on Research Square: Chen-Ku CH, de Sucre PG, Vinocour-Fournieri M, et al.: Diabetes prescription patterns in Costa Rica and Panama among patients switching to a second-line medication. Evidence from the DISCOVER real-world diabetes registry. December 10, 2020. (DOI: 10.21203/rs.3.rs-119077/v1)

## Materials and methods

DISCOVER is an observational diabetes registry of patients switching from first- to second-line glucose-lowering medication (mono or combination) in 38 countries across six continents [11]. Patients were recruited from December 2014 to June 2016 for a three-year follow-up after being prescribed a second-line medication. In this study, we report baseline data for Costa Rica and Panama. Enrolled patients underwent clinical assessments and received standard medical care as determined by their treating physician. Physicians invited to participate were selected across different specialties and healthcare settings to ensure that the findings would be representative of diabetes management in each country. The sponsor selected the sites and investigators in each country based on patient volume and the investigator’s experience in clinical trials. In Costa Rica, only private centers were selected given that the public counterparts at the time of enrollment were in the process of approval of new regulations regarding clinical studies. In Panama, three centers were invited to participate but one (private) did not recruit any patients. Therefore, this study presents data from six private centers in Costa Rica and two (one public and one private) in Panama. 

Patient participation was on a voluntary basis and they could withdraw from the registry at any time without compromising their treatment. The protocol was approved by the Scientific Ethics Committee of Costa Rica Institute for Clinical Research (Comité Ético Científico Instituto Costarricense de Investigaciones Clínicas) in Costa Rica and by the Research Bioethics Committee of the Gorgas Memorial Institute for Health Studies (Comité de Bioética de la Investigación del Instituto Conmemorativo Gorgas de Estudios de la Salud) in Panama. All participating patients provided signed informed consent.

To be included, patients must be at least 18 years old and have type 2 diabetes. In addition, they must require second-line (add-on or switching analyzed together) after first-line oral medication. These are any antidiabetic drugs either monotherapy or fixed-dose combination started after diabetes diagnoses. Patients with type 1 diabetes, pregnancy, on chemotherapy or steroids, undergoing dialysis or renal transplant recipients were excluded. Those taking alternative diabetes treatments (e.g., herbal remedies), insulin, or an injectable agent as first-line treatment, initiating dual therapy after two different lines of monotherapy were also excluded. Other exclusion criteria included enrollment in any interventional trial and if the recruiting physician considered that the patient had any other circumstance or condition that could significantly decrease the likelihood of follow-up [11].

Demographic and anthropometric data, laboratory results, past medical history, medications, and healthcare provider type were collected. Waist circumference, weight, and height were measured by the investigator in a standardized manner. Normal weight, overweight, and obesity were classified according to the respective body mass index (BMI) categories. Blood tests included: glycosylated hemoglobin (HbA1c), total, high-density lipoprotein (HDL), and low-density lipoprotein (LDL) cholesterol. Treatment targets were defined as HbA1c < 7% (53 mmol/mol), blood pressure (BP) < 130/90 mm Hg, and LDL cholesterol < 70 mg/dl. Regarding treatment, choice of first- and second-line were recorded and the reasons to switch therapy. All data were collected from the patients’ charts (electronically when available). 

All patients in the registry from Costa Rica and Panama were included in the analysis (therefore no sample size estimation was required). Descriptive statistics were used to summarize demographic variables, medications, changes in HbA1c, blood glucose, lipid profile, BMI, waist circumference, blood pressure and complications, hospitalizations, and hypoglycemia events. Analysis was done comparing each country using chi-square (Fisher exact test when appropriate) and ANOVA (Mann-Whitney U test when not normally distributed) in Stata software (College Station, TX: StataCorp LLC).

## Results

All 219 patients spread across eight different sites (six in Costa Rica, two in Panama) were analyzed; 127 in Costa Rica and 92 in Panama. Most (five in Costa Rica, one in Panama) of the sites were specialized in diabetes care with endocrinologists/diabetologists. 

Most of the patients were female (61%) and the mean age was 58.7 (± 12.8) years (Table 1). Waist circumference and BMI were significantly different by country. The former was, on average, significantly higher in Costa Rica compared to Panama. Likewise, the prevalence of obesity was 72% in Costa Rica and 26% in Panama. About two-thirds of patients in each country presented hypertension, and the prevalence of hyperlipidemia was significantly higher in Costa Rica compared to Panama. Intake of lipid-lowering medications was also significantly higher in Costa Rica (Table 1).

**Table 1:** Baseline demographic characteristics of patients in the DISCOVER-CA** *As reported by the treating physician **Costa Rica and Panama IQR: interquartile range

	Total	Costa Rica	Panama	
	n (%)	n (%)	n (%)	p-Value
Total	219 (100)	127 (57.9)	92 (42.0)	
Male	86 (39.30)	55 (43.3)	31 (33.7%)	
Age (mean, SD)	58.7 ± 12.8	57.0 ± 13.6	61.1 ± 11.4	0.02
Year since diagnosis (median, IQR)	4.91 (1.24-9.98)	5.54 (1.32-8.02)	7.83 (1.13-10.74)	<0.001
Waist circumference (cm)	99.5 ± 16.6	105.5 ± 16.6	90.8 ± 12.2	<0.001
Body mass index				0.0004
≤25	25 (15.92)	10 (9.3)	15 (30.0 )	
25 to <30	61 (38.85)	39 (36.4)	22 (44.0)	
30≥	71 (45.22)	58 (54.2 )	13 (26.0 )	
Hypertension*	131 (59.8)	72 (56.7)	59 (64.1)	0.2
Hyperlipidemia*	85 (38.80)	58 (45.7)	27 (29.3)	0.01
Concomitant medications				
Antihypertensives	134 (61.20)	81 (63.8)	53 (57.6)	0.3
Lipid-lowering	103 (47)	70 (55.1)	33 (35.9)	0.004
Antiplatelet	33 (15.10)	21 (16.5)	12 (13.0)	0.4

In the first visit, about one-third of patients in Costa Rica, and nearly one-quarter in Panama were lacking an HbA1c. For those with available data, the number of patients with high blood pressure at physical examination was significantly higher in Panama (51%) compared to Costa Rica (25.8%). Most patients in both countries did not have any cholesterol results. Regarding total cholesterol, HDL, and LDL, the majority of patients had high levels; with there being no difference between both countries (Table 2).

**Table 2:** Chronic disease risk factors, screening tests, and abnormal results in DISCOVER-CA* *Costa Rica and Panama HbA1c: glycosylated hemoglobin; HDL: high-density lipoprotein; LDL: low-density lipoprotein

	Costa Rica (n=127)		Panama (n=92)		
	Missing n (%)	n (%)	Missing n (%)	n (%)	p-Value
HbA1c>7% (53 mmol/mol)	25 (32.5)	67 (65.6)	31 (23.5)	42(68.8)	0.6
Blood pressure >130/90 mm Hg	15 (16.8)	29 (25.8)	14 (12.2)	40 (51.3)	0.0003
Total cholesterol >180 mg/dL	64 (69.6)	26(41.3)	56 (50.4)	24 (66.7)	0.01
HDL <40M/50W mg/dL	66 (72.5)	34 (55.7)	59 (52.5)	20 (60.6)	0.2
LDL >70 mg/dL	68 (72.5)	50 (84.7)	57 (52.5)	28 (80.0)	0.3

Reasons to switch to second-line medication did not differ by country. About two-thirds switched due to lack of efficacy and about 20% due to side effects (Table 3). Furthermore, switching medication due to a hypoglycemic event was rare. To choose the second-line medication, there were significant differences between countries. In Costa Rica, the medication effect on promoting weight loss was the most common, followed by efficacy and tolerability. In Panama, it was efficacy followed by tolerability and weight. In both countries, the cost was not a common denominator to choose the second-line medication. 

**Table 3:** Reasons to switch from first- to second-line and choosing second-line therapy in the DISCOVER-CA* *Costa Rica and Panama

	Total	Costa Rica	Panama	
	n (%)	n (%)	n (%)	p-Value
Reasons to switch from fist- to second-line				
Lack of efficacy	146 (66.7)	88 (69.3)	58 (63)	0.33
Hypoglycemic event	5 (2.30)	2 (1.6)	3 (3.3)	0.4
Weight gain	13 (5.90)	5 (3.9)	8 (8.7%)	0.1
Side effect	39 (17.80)	24(18.9)	15 (16.3)	0.6
Affordability	3 (1.40)	3 (2.4)	0 (0)	
Reasons for choosing second-line				
Efficacy	96 (43.80)	46 (36.2)	50 (54.3)	0.01
Tolerability	58 (26.50)	39 (30.7)	19 (20.7)	0.1
Weight	73 (33.3)	62 (48.8)	11 (12.0)	<0.00001
Hypoglycemia	38 (17.40)	27 (21.3)	11 (12.)	0.07
Patient request	1 (0.50)	0 (0)	1 (1.1)	
Convenience	23 (10.50)	19 (15)	4 (4.3)	0.01
Access	23 (10.50)	12 (9.4)	11 (12)	0.5
Cost	9 (4.10)	7 (5.5)	2 (2.2)	0.2

The most frequently prescribed first-line medication was metformin (MET) (Table 4). While sulphonylureas (SU) were the second most commonly prescribed medicine in Panama, they only accounted for 3% in Costa Rica. The second most common scheme used in Costa Rica was a combination of MET and dipeptidyl peptidase-4 inhibitor (iDPP4) (only 2% in Panama). The most common combination used as first-line in Panama was MET plus SU. Regarding second-line medication, while 11 different medications were prescribed in Costa Rica, only seven were in Panama. The MET plus iDPP4 combination was the most commonly used in both countries. Although this accounted for most cases in Panama (58.7%), it was only prescribed in 24% of patients in Costa Rica. MET plus sodium-glucose transport protein-2 inhibitor (iSGLT2) accounted for another 20% of the prescriptions in Costa Rica. Only one patient started insulin (as monotherapy) on second-line medication and none in Panama.

**Table 4:** First- and second-line medications in DISCOVER-CA* *Costa Rica and Panama iDPP4: dipeptidyl peptidase-4 inhibitor; SGLT2: sodium-glucose cotransporter-2

	Costa Rica		Panama	
Medication type	First	Second	First	Second
Metformin	75 (59.1)	2 (1.6)	67 (72.8)	3 (3.3)
Sulphonylureas	4 (3.1)		13 (14.1)	1 (1.1)
iDPP4	15 (11.8)	11 (8.7)		13 (14.1)
SGLT2 inhibitor		14 (11.0)		
Other mono	1 (0.8)			
Metformin + Sulphonylureas	7 (5.5)	9 (7.1)	10 (10.1)	3 (3.3)
Metformin + iDPP4	23 (18.1)	31 (24.4)	2 (2.2)	54 (58.7)
Metformin + SGLT2 inibitor		26 (20.5)		
Other dual	2 (1.6)	7 (5.5)		7 (7.6)
Metformin + Sulphonylureas + iDPP4		3 (2.4)		9 (9.8)
Other three		22 (17.3)		
Other four		1 (0.8)		
Insulin		1 (0.8)		

## Discussion

According to our findings, the most common second-line medication prescribed in the selected centers from Costa Rica and Panama was a combination of MET plus iDPP4, followed by MET plus iSGLT2s in Costa Rica, and iDPP4 monotherapy in Panama. To the extent of our knowledge, this is the first study to document diabetes medication switching patterns in a real-world setting in Central America. 

DISCOVER also includes data on cardiovascular disease (CVD) risk factors including blood pressure and cholesterol levels [11]. In our sample, most patients had a background of hypertension, and up to 50% had high blood pressure upon clinical examination. Even though the prevalence of hypertension was lower in Costa Rica, the use of antihypertensive drugs was higher when put against Panama. This may be due, in part, to the hypertension report as a diagnosis could be lower in the former compared to the latter. Regarding cholesterol, nearly half our sample was taking a lipid-lowering drug, and these were more frequent in Costa Rica. Prevalence of hypertension in patients with diabetes has been reported between 60% (Thailand and Mexico) and 80% (Scotland) and hypercholesterolemia between 35% (Mexico) and over 55% (Scotland, Iran, England, Colombia, Thailand, and United States) [12]. Therefore, CVD risk factors among patients with diabetes in DISCOVER Costa Rica and Panama appear to be nestled in the lower range compared to other countries [12]. This finding deserves additional population-based data to be confirmed. 

The number of patients with missing data in both countries should be a matter of concern. Even for HbA1c, not all patients had baseline measurements. Why patients lack this information merits further research. However, it might be due that some tests were performed outside of the three-month period allowed to be included in DISCOVER [11]. Regarding blood pressure, nearly 20% of patients did not have the data in the aforementioned chart. Missing data has also been reported elsewhere. Blood pressure was not recorded in 7.7% of patients with diabetes in Colombia, 16% in England, 0.5% in Iran, 3% in México, 15.1% in Scotland, 0.34% in Thailand, and 10.5% in the United States. Cholesterol results were not available in 1.15%, 33.3%, 1.4%, 10%, 32.8%, 0.54% and 22.9%, respectively [12]. Strikingly, lower-income countries have a higher rate of reporting blood pressure and blood cholesterol compared to higher-income ones. Even if this represents a failure to document in the medical record, it should be subject of attention as blood pressure and cholesterol control are interventions that decrease mortality in patients with diabetes. 

Glycemic control (through HbA1c) was far from optimal despite the use of oral antidiabetic agents. Approximately one-third of patients in Costa Rica and Panama had HbA1c levels below 7% compared to 15.6% reported in Latin America [10]. When using HbA1c as a marker of glucose control our results in Costa Rica and Panama are similar to those reported on a systematic review in Latin America [7] and are consistent with previous reports [2,13]. In the United States, 26% of patients met HbA1c targets. In Colombia, 27% of males and 24% of females met HbA1c targets [12]. Our registry includes patients switching to second-line medication and thus, it is expected that most of them would have high HbA1c. However, the results yield that there is a delay in the initiation of second-line therapies and clinical inertia, which are worldwide recognized issues. 

First-line medication choice was in accordance with most evidence-based 2014 guidelines that include MET alone or in combination with DDP4 inhibitors [4,14,15]. Additionally, SU alone or in combination was prescribed in 15.5% of patients [14,15]. According to our findings, the choice of first-line medication in DISCOVER Costa Rica and Panama was made based on 2014 guidelines. 

When choosing second-line medications, better efficacy, promoting weight loss, tolerability, and lower risk of hypoglycemia were the most commonly reported reasons in DISCOVER Costa Rica and Panama. Weight gain was an important factor in one-third of the cases and this seems logical given the high prevalence of overweight or obesity in our registry (overweight or obesity in our sample ranged from 70% to 90%). In Costa Rica, the most commonly prescribed medications were MET combined with iDPP4 followed by MET combined with iSGLT2. In Panama, MET combined with iDPP4 and iDPP4 monotherapy was the most common. In Costa Rica, iSGLT2 and iDPP4 are available only in the private system (where DISCOVER patients are recruited from) at a lower cost than in high-income countries and similarly priced. According to the DISCOVER registry, the cost is not one of the reasons for choosing the second-line medication (even in Costa Rica where patients usually pay out-of-pocket). In Panama, iDPP4 are available in the public health system and iSGLT2 only in the private system. This can explain, in part, the higher prescription of iDPP4. Under similar circumstances (access and healthcare system), as in Panama, iDPP4 are preferred over SU. Despite guideline recommendations in 2016, glucagon-like peptide-1 (GLP1) receptor agonists were not prescribed [16]. This might be due to the fact that at the time when patients were enrolled in DISCOVER, GLP1 receptor agonists and thiazolidinediones were not readily available. Therefore, our findings suggest that the main drivers for second-line prescription are efficacy, side effects, and promoting weight loss. Drug availability and access limit the number of available options.

According to our findings, second-line prescription patterns differ from those in high-income countries. In the United States, SU is still the most widely prescribed followed by iDPP4 and iSGLT2 [17]. The difference in the use of SU might be explained by the healthcare system and pricing, given that the price of SU is much lower than iDPP4 or iSGLT2 in the United States. In Denmark, Norway, and Sweden, SU was the most common second-line and iDPP4 in Finland [18]. During 2014-2016, in Korea, iDPP4 was the most frequently prescribed, followed by SU and iSGLT2. Choice of second-line medication might also be determined by physician specialty (internists tended to choose newer agents) [19]. Also, iSGLT2 and iDPP4 were most often prescribed in tertiary hospitals compared to clinics as well as in urban settings compared to rural ones [19]. Recently, this change in prescription patterns has also been noticed and mentioned in the United Kingdom, where iDPP4 use almost doubled as second-line from 2010 to 2017 in a cohort of 81,532 patients, becoming the most commonly prescribed second-line medication after 2016. This change was accompanied by a decrease in weight, blood pressure, and hypoglycemia rates [20].

Our study has strengths and limitations. To the best of our knowledge, in Central America, the DISCOVER Registry is the first real-world description of diabetes prescription outside of clinical trials. In addition, at least in some Central American centers. DISCOVER provides insights into the reasons leading to change from first- to second-line medications. Small sample size, recruitment from only private centers in Costa Rica, and the fact that patients voluntarily enrolled in the registry might introduce “healthy volunteer” selection bias. Therefore, our findings might not be generalizable country-wide. Furthermore, we only document prescriptions and not adherence to medications. Finally, lifestyle modifications (e.g., physical activity, diet) that influence response to treatment are not considered. Despite these limitations, DISCOVER provides data about prescription patterns and the reasons to switch from first to second-line antidiabetic agents in selected centers from two Central American countries.

## Conclusions

In conclusion, metformin was the most common first-line therapy, and metformin plus DPP4 inhibitors were the most commonly prescribed second-line therapy. Our findings provide evidence that when switching to second-line medications in Costa Rica and Panama in the DISCOVER registry, efficacy, side effects, and weight loss appear to be the main drivers as opposed to costs. Furthermore, medication choice agreed with the guidelines available at the time. 
